# MicroRNA, Myostatin, and Metabolic Rate Depression: Skeletal Muscle Atrophy Resistance in Hibernating *Myotis lucifugus*

**DOI:** 10.3390/cells13242074

**Published:** 2024-12-16

**Authors:** W. Aline Ingelson-Filpula, Sarah A. Breedon, Kenneth B. Storey

**Affiliations:** 1 Department of Biology, Carleton University, 1125 Colonel By Drive, Ottawa, ON K1S 5B6, Canada; alineingelsonfilpula@cmail.carleton.ca (W.A.I.-F.); kenstorey@cunet.carleton.ca (K.B.S.); 2 Institute of Biochemistry, Carleton University, 1125 Colonel By Drive, Ottawa, ON K1S 5B6, Canada

**Keywords:** little brown bat, atrophy, myostatin, signaling, torpor, insulin, cytoskeleton, proteolysis, degradation

## Abstract

Little brown bats (*Myotis lucifugus*) cluster in hibernacula sites over winter, in which they use metabolic rate depression (MRD) to facilitate entrance and exit of hibernation. This study used small RNA sequencing and bioinformatic analyses to identify differentially regulated microRNAs (miRNAs) and to predict their downstream effects on Gene Ontology (GO) and Kyoto Encyclopedia of Genes and Genomes (KEGG) terms in the skeletal muscle of torpid *M. lucifugus* as compared to euthermic controls. We observed a subset of ten miRNAs whose expression changed during hibernation, with predicted functional roles linked to cell cycle processes, downregulation of protein degradation via ubiquitin-mediated proteolysis, downregulation of signaling pathways, including MAPK, p53, mTOR, and TGFβ, and downregulation of cytoskeletal and vesicle trafficking terms. Taken together, our results indicate miRNA regulation corresponding to both widely utilized MRD survival strategies, as well as more hibernation- and tissue-specific roles in *M. lucifugus*, including skeletal muscle atrophy resistance via myostatin inhibition and insulin signaling suppression.

## 1. Introduction

Like many animals living in regions that experience cold winters, the little brown bat (*Myotis lucifugus*) is susceptible to decreased temperatures and food scarcity over the winter months. In order to survive these harsh conditions, the bats increase their body mass by ~30% going into winter to build up lipid stores and then hibernate in caves or other hibernacula sites for weeks to months at a time [1,2]. Mammalian hibernation is typically characterized by multiple torpor-arousal periods over a period of several months. During torpor, the body temperature (T_b_) of *M. lucifugus* falls to near ambient temperatures, their metabolic rate drops to ~1–1.5% of euthermic values, and there is a marked decrease in the heart and respirometry rates [3]. In the brief interbout arousal phase, the T_b_ rises back to ~37 °C, and the normal metabolic functions resume before the T_b_ falls again and another torpor bout begins [3]. Little brown bats, like other hibernators, use metabolic rate depression (MRD) to facilitate shifts from normometabolic states to hypometabolic states [4,5]. During MRD, non-essential and energetically expensive processes such as cell replication and protein synthesis are downregulated to conserve endogenous cellular energy, while pro-survival pathways are prioritized to protect cells and tissues from damage [4,5].

Achieving this metabolic suppression without suffering ill effects upon reversal requires a vast interplay of regulatory mechanisms at all cellular levels, including transcriptional, translational, and epigenetic. One form of regulation that has been of much recent interest in MRD research is post-transcriptional gene silencing via microRNA (miRNA). These miRNAs are short (21–24 nt), noncoding, single-stranded RNA, which target mRNA transcripts for degradation or translational suppression [6]. miRNA are potent regulators of gene expression, given that one miRNA can target multiple mRNAs and one mRNA can likewise be silenced by multiple miRNAs [6]. Many studies have indicated that miRNA regulation is an integral mechanism of MRD in various animals that survive extreme environmental stresses [1,4,7,8,9,10,11,12,13,14,15]. Indeed, a previous study on *M. lucifugus* found that hibernation-induced miRNA expression in the brain targeted focal adhesion and axon guidance pathways [16]. Additionally, another study measured the expression of ten miRNAs in *M. lucifugus* skeletal muscle and predicted that the differentially expressed miRNAs may serve myoprotective roles [17]. It is well-known that mammals experience skeletal muscle atrophy during periods of prolonged immobility, yet mammalian hibernators awaken in the spring with little to no sign of atrophy [18,19,20]. Thus, the regulation of miRNA-mediated gene silencing is likely critical for skeletal muscle maintenance during hibernation through myoprotective and anti-apoptotic functions.

We sought to build upon the previous study by Kornfeld et al. [17] through the use of next-generation sequencing to compare the skeletal muscle miRNA profiles of euthermic and torpid *M. lucifugus* and determine which miRNAs are differentially expressed in torpor. Gene Ontology (GO) and Kyoto Encyclopedia of Genes and Genomes (KEGG) pathway analyses were also used to predict which cellular pathways are most affected by these changes in miRNA expression. Overall, it was hypothesized that there would be miRNA-mediated downregulation of energetically expensive processes, whereas upregulation may tend towards pro-survival pathways critical to the survival of torpor and preparation for return to a normometabolic state, including the upregulation of myoprotective processes. This research further advances our understanding of the regulatory mechanisms underlying hibernation in *M. lucifugus* and the role miRNAs play in MRD.

## 2. Materials and Methods

### 2.1. Animal Collection

*Myotis lucifugus* were collected by D.W. Thomas in November 1999, as previously described [21]. The collection was permitted by the Ministère des Ressources Naturelles et de la Faune du Québec, and the subsequent animal experimentation followed protocols approved by the animal care committee of the Université de Sherbrooke, in compliance with the Canadian Council on Animal Care guidelines. After transport, half of the bats were kept awake in a warm room (23–24 °C), and the remaining bats were placed in a cold room (5 °C) to re-enter torpor. The bats in the cold room took ~12 h to return to full torpor. Temperature-sensitive radio transmitters (SRX-400, Lotek Engineering, Newmarket, ON, Canada) were attached to the interscapular region of the bats to monitor skin temperature every 5 min, which was used to approximate T_b_ and confirm that the bats were continually torpid at the time of sampling [16,22]. The euthermic bats were euthanized after ~48 h of T_b_ 35–37 °C, and the torpid bats were euthanized after ~36–38 h of torpor with T_b_ 5–6 °C. Following euthanasia, tissues were rapidly dissected, flash-frozen in liquid nitrogen, and then stored at −80 °C until use.

### 2.2. RNA Extraction

RNA was extracted from the skeletal muscle (~50 mg) of euthermic and torpid *M. lucifugus* (*n* = 4 for both conditions) as previously described [10]. The tissues were crushed under liquid nitrogen, homogenized in 1 mL of TRIzol reagent (Invitrogen, Carlsbad, CA; Cat. # 15596-018) with a Polytron homogenizer (PT 1200; Kinematica, Werkstrasse, Switzerland), and 200 µL of chloroform was added. The samples were then centrifuged at 10,000 rpm for 15 min (4 °C). The upper aqueous phase containing RNA was mixed with 500 µL of 2-propanol and allowed to incubate at room temperature for 10 min to allow the RNA to precipitate. The RNA was then pelleted via centrifugation at 10,000 rpm for 15 min (4 °C), after which the supernatant was discarded. The pellets were washed twice with ethanol (70%) and left to air-dry for 10–15 min. RNase-free water (50 µL) was used to resuspend the RNA pellets, and then the concentration and purity were measured using a BioTek Take3 and a PowerWave HT microplate spectrophotometer (Winooski, VT, USA). The RNA integrity was confirmed with an OD 260/280 ratio of ~2.0 and via the presence of 28S and 18S rRNA bands with agarose gel (1%) electrophoresis.

### 2.3. Small RNA Sequencing

The RNA samples from the skeletal muscle of euthermic and torpid *M. lucifugus* were sequenced by the Center for Applied Genomics at the SickKids Hospital (Toronto, ON, Canada). An Agilent Bioanalyzer 2100 system (Agilent Technologies, Santa Clara, CA, USA) was used to confirm RNA quality before miRNA library construction. Small RNA cDNA libraries were assembled and validated as previously described [23], and sequenced with an Illumina HiSeq 2500 platform (San Diego, CA, USA). All raw small-RNA data can be found on the Sequence Read Archive (SRA) database (BioProject ID: PRJNA1100517).

### 2.4. Read Processing

The raw read data were processed using the RBioMIR pipeline (https://github.com/jzhangc/git_RBioMIR, accessed on 12 November 2023), as previously described [24]. Broadly, cutadapt (v.0.10.1) filtered out low-quality reads [25], and FastQC (http://www.bioinformatics.babraham.ac.uk/projects/fastqc/) validated small RNA lengths (~25 nt) [26]. Bowtie (http://bowtie.cbcb.umd.edu) isolated mature miRNA sequences from other non-miRNA small RNAs (tRNA, rRNA, piRNA, snRNA, and snoRNA) via a negative reference file consisting of total Rfam (https://rfam.xfam.org) [27] and piRNABank databases [28] and aligned the remaining reads to total mature miRNA sequences from miRBase (http://www.mirbase.org/) [29], with parameters set to perfect seed sequence matches and a seed sequence length of 20 nt [30]. Samtools (https://www.htslib.org/) was then used to sort and count reads that aligned to mature miRNA sequences [31]. miRNA reads with >4 reads were removed, and read counts were normalized as previously described using voom [24,32].

### 2.5. Differential Expression Analysis

Differential expression of miRNAs between euthermic and torpid animals was determined using the limma R package (https://bioconductor.org/packages/release/bioc/html/limma.html) [33]. Statistically significant differentially expressed miRNAs had a false discovery rate (FDR)-corrected *p*-value < 0.05 and fold-change ≥ 1.5.

### 2.6. Gene Set Enrichment Analysis

The RBiomirGS R package (https://github.com/jzhangc/git_RBiomirGS, accessed on 12 November 2023) was used for gene set analyses to determine significantly enriched GO and KEGG pathways [34,35,36]. RBiomirGS enumerates miRNA:mRNA interactions and then calculates a miRNA score (*S_microRNA_*) and a mRNA score (*S_mRNA_*) to quantify potential interactions between miRNAs and their mRNA targets [37]. The calculated *S_mRNA_* of mRNAs is then used to identify significantly enriched gene sets (FDR-adjusted *p*-value ≥ 0.05) and calculate the model coefficients of each GO and KEGG term. Negative model coefficients indicate that the term is downregulated due to increased negative regulation by miRNA, and positive model coefficients indicate upregulation via decreased miRNA regulation during torpor as compared with arousal. Estimated model coefficients and standard errors are calculated using a logistic regression-based method and applied to determine statistical significance [34].

## 3. Results

### 3.1. Small RNA Sequencing Summary

The mean raw reads from the euthermic and torpid *M. lucifugus* skeletal muscle were 7,666,912 ± 274,700 and 7,644,381 ± 733,010, respectively. Trimming and quality filtering yielded 2,606,847 ± 414,774 control reads and 2,431,873 ± 260,932 torpid reads. Following negative filtering, 2,587,613 ± 420,441 reads in euthermic animals aligned to 142 mature miRNA sequences, whereas 2,405,990 ± 261,905 reads aligned in torpid animals.

### 3.2. Differential Expression of microRNA in Response to Torpor

**Figure 1:**
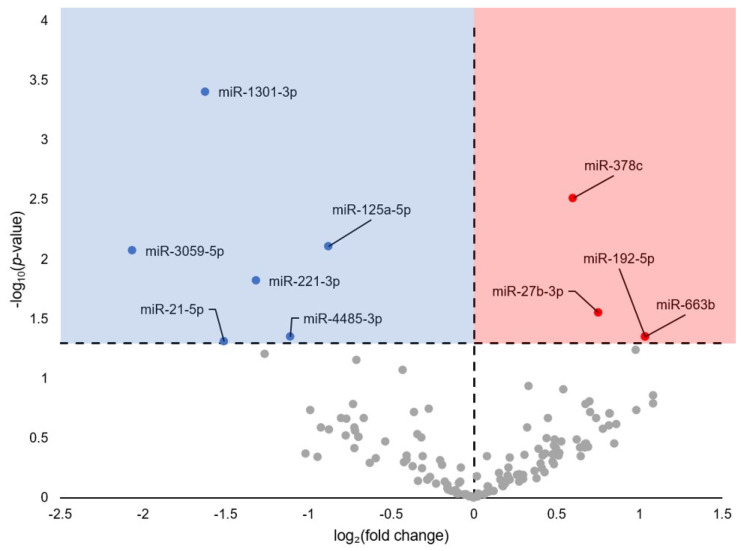
Differentially expressed miRNA in euthermic vs. hibernating *Myotis lucifugus* skeletal muscle. Fold-change thresholds were set to ± log_2_1.5 with a false discovery rate (FDR)-adjusted *p*-value < 0.05. Blue markers indicate significantly downregulated miRNA and red markers indicate significantly upregulated miRNA. Grey markers are miRNAs that did not pass the fold-change and *p*-value thresholds.

In total, 142 mature miRNAs aligned with the filtered reads, of which ten had a statistically significant (FDR-adjusted *p*-value < 0.05) change in expression during torpor as compared to euthermic controls (Figure 1; Supplementary File S1). Specifically, six were downregulated (miR-1301-3p, miR-125a-5p, miR-3059-5p, miR-221-3p, miR-4485-3p, miR-21-5p; blue circles) and four were upregulated (miR-378c, miR-27b-3p, miR-192-5p, miR-663b; red circles).

### 3.3. Gene Set Enrichment Analysis

**Figure 2:**
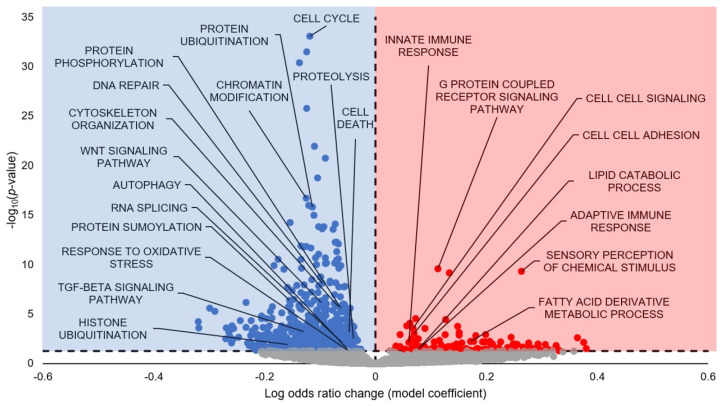
Gene Ontology GO Biological Processes predicted from torpor-induced miRNA in euthermic vs. hibernating *M. lucifugus* skeletal muscle. All significantly enriched terms had an FDR-adjusted *p*-value < 0.05. Blue markers (negative model coefficients) indicate upregulation of miRNA targeting of the genes and resultant term downregulation. Red markers (positive model coefficients) indicate downregulation of miRNA targeting of the genes and resultant term upregulation. Grey markers indicate terms that did not pass the *p*-value thresholds.

GO Biological Processes (GO BP) analysis predicted 654 terms to be statistically significantly enriched (FDR-adjusted *p*-value < 0.05), with 112 upregulated and 452 downregulated (Figure 2; Supplementary File S2). A selection of the terms predicted to be statistically significantly upregulated include Innate Immune Response, G-Protein Coupled Receptor Signaling Pathway, Cell-Cell Signaling, Cell-Cell Adhesion, Lipid Catabolic Process, Adaptive Immune Response, Sensory Perception of Chemical Stimulus, and Fatty Acid Derivative Metabolic Process. A selection of the terms predicted to be statistically significantly downregulated include Cell Cycle, Protein Ubiquitination, Protein Phosphorylation, DNA Repair, Chromatin Modification, Cytoskeleton Organization, WNT Signaling Pathway, Autophagy, RNA Splicing, Proteolysis, Cell Death, Protein SUMOylation, Response to Oxidative Stress, TGF-β Signaling Pathway, and Histone Ubiquitination.

**Figure 3:**
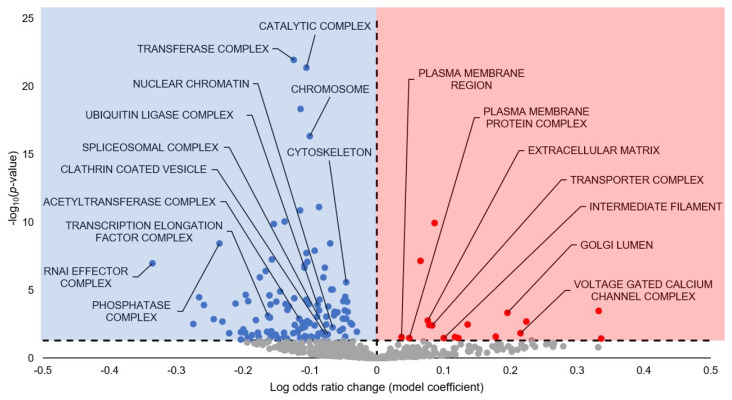
Gene Ontology GO Cellular Compartment predicted from torpor-induced miRNA in euthermic vs. hibernating *M. lucifugus* skeletal muscle. All significantly enriched terms had an FDR-adjusted *p*-value < 0.05. Blue markers (negative model coefficients) indicate upregulation of miRNA targeting of the genes and resultant term downregulation. Red markers (positive model coefficients) indicate downregulation of miRNA targeting of the genes and resultant term upregulation. Grey markers indicate terms that did not pass the *p*-value thresholds.

For the GO Cellular Compartment (GO CC) analysis, 136 terms are significantly enriched, with the majority being downregulated (Figure 3; Supplementary File S3). A selection of the terms predicted to be statistically significantly upregulated include Plasma Membrane Region, Plasma Membrane Protein Complex, Extracellular Matrix, Transporter Complex, Intermediated Filament, Golgi Lumen, and Voltage Gated Calcium Channel Complex. A selection of the terms predicted to be statistically significantly downregulated include Catalytic Complex, Transferase Complex, Chromosome, Cytoskeleton, Nuclear Chromatin, Ubiquitin Ligase Complex, Spliceosomal Complex, Clathrin Coated Vesicle, Acetyltransferase Complex, Transcription Elongation Factor Complex, RNAi Effector Complex, and Phosphatase Complex.

**Figure 4:**
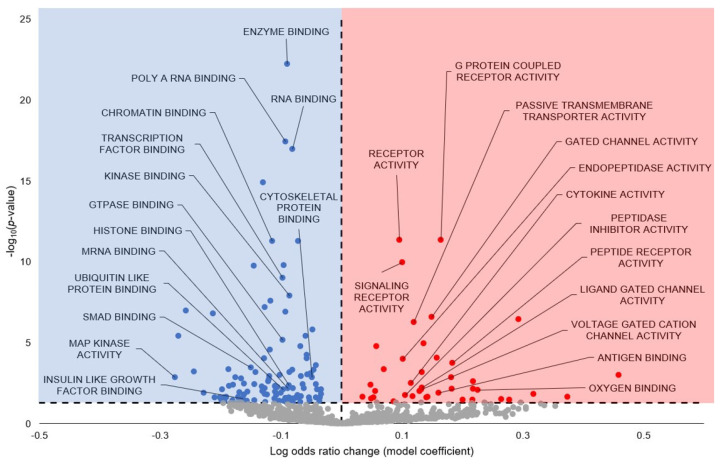
Gene Ontology GO Molecular Function predicted from torpor-induced miRNA in euthermic vs. hibernating *M. lucifugus* skeletal muscle. All significantly enriched terms had an FDR-adjusted *p*-value < 0.05. Blue markers (negative model coefficients) indicate upregulation of miRNA targeting of the genes and resultant term downregulation. Red markers (positive model coefficients) indicate downregulation of miRNA targeting of the genes and resultant term upregulation. Grey markers indicate terms that did not pass the *p*-value thresholds.

In GO Molecular Function (GO MF), 151 terms are statistically significant—of those, 111 are downregulated, and 40 are upregulated (Figure 4; Supplementary File S4). A selection of the terms predicted to be statistically significantly upregulated include G-Protein Coupled Receptor Activity, Receptor Activity, Signaling Receptor Activity, Passive Transmembrane Transporter Activity, Gated Channel Activity, Endopeptidase Activity, Cytokine Activity, Peptidase Inhibitor Activity, Peptide Receptor Activity, Ligand Gate Channel Activity, Voltage Gated Cation Channel Activity, Antigen Binding, and Oxygen Binding. A selection of the terms predicted to be statistically significantly downregulated include Enzyme Binding, Poly(A) RNA Binding, RNA Binding, Chromatin Binding, Transcription Factor Binding, Kinase Binding, GTPase Binding, Histone Binding, mRNA Binding, Ubiquitin-Like Protein Binding, SMAD Binding, MAP Kinase Activity, Insulin-Like Growth Factor Binding, and Cytoskeletal Protein Binding.

**Figure 5:**
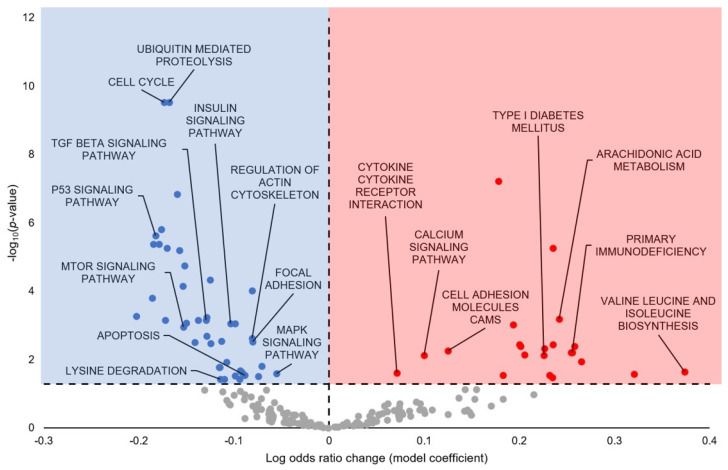
KEGG Pathway Analysis predicted from torpor-induced miRNA in euthermic vs. hibernating *M. lucifugus* skeletal muscle. All significantly enriched terms had an FDR-adjusted *p*-value < 0.05. Blue markers (negative model coefficients) indicate upregulation of miRNA targeting of the genes and resultant term downregulation. Red markers (positive model coefficients) indicate downregulation of miRNA targeting of the genes and resultant term upregulation. Grey markers indicate terms that did not pass the *p*-value thresholds.

The KEGG pathway analysis predicted 64 pathways to be differentially regulated during hibernation, with 22 upregulated and 42 downregulated (Figure 5; Supplementary File S5). A selection of the terms predicted to be statistically significantly upregulated include Arachidonic Acid Metabolism, Type I Diabetes Mellitus, Primary Immunodeficiency, Valine Leucine and Isoleucine Biosynthesis, Cell Adhesion Molecules (CAMs), Calcium Signaling Pathway, and Cytokine–Cytokine Receptor Interaction. A selection of the terms predicted to be statistically significantly downregulated include Ubiquitin-Mediated Proteolysis, Cell Cycle, TGF-β Signaling Pathway, P53 Signaling Pathway, mTOR Signaling Pathway, Insulin Signaling Pathway, Regulation of Actin Cytoskeleton, Focal Adhesion, MAPK Signaling Pathway, Apoptosis, and Lysine Degradation.

## 4. Discussion

### 4.1. Cell Cycle Processes Downregulated During Hibernation

A vast majority of statistically significant terms in GO BP, GO CC, and GO MF were linked to cell cycle-related processes, along with KEGG, which had Cell Cycle as a statistically significant downregulated term (Figure 2, Figure 3, Figure 4 and Figure 5; Supplementary Files S2–S5). In GO BP, 30 statistically significant terms contained the term ‘cell cycle’, with a further 14 containing ‘mitotic’ as a descriptor (Supplementary File S2). All of these terms had negative model coefficients, signifying that miRNA influence is predicted to downregulate these collective processes during torpor in *M. lucifugus* skeletal muscle. The cell cycle is an energetically expensive process, and as such, we have observed cell cycle downregulation during hypometabolic states in a vast array of species [38]. Furthermore, miRNAs themselves have been widely reported to downregulate cell cycle and proliferation in response to environmental stress in many different animals [7,9,12,23,39,40,41]. Therefore, our observation of cell cycle quiescence in hibernating *M. lucifugus* skeletal muscle is well-supported in the literature and further backs this as a conserved hypometabolic strategy during MRD.

### 4.2. Suppression of Protein Degradation May Link to Myostatin Inhibition

Many significant GO terms with a negative model coefficient were related to cellular degradation processes, such as Proteolysis, Positive Regulation of Proteolysis, Protein Ubiquitination, and Histone Ubiquitination (GO BP; Figure 2; Supplementary File S2); Ubiquitin Ligase Complex, Cullin Ring Ubiquitin Ligase Complex, and Spliceosomal Complex (GO CC; Figure 3; Supplementary File S3); and Ubiquitin-Like Protein Binding, Thiol-Dependent Ubiquitin-Specific Protease Activity, Ubiquitin-Like Protein Specific Protease Activity, Ubiquitin-Like Protein Conjugating Enzyme Activity, Ubiquitin-Like Protein Transferase Activity, Ubiquitin-Like Protein Ligase Activity, Ubiquitin-Like Protein Ligase Binding, Ubiquitin-Like Protein Conjugating Enzyme Binding (GO MF; Figure 4; Supplementary File S4). Finally, KEGG displayed Ubiquitin-Mediated Proteolysis and Lysine Degradation as statistically significant with negative model coefficients (Figure 5; Supplementary File S5).

Ubiquitin-mediated proteolysis is a key method of protein degradation in eukaryotic cells, with ubiquitin conjugation being the most prevalent post-translational modification [42]. Broadly, ubiquitin is conjugated to protein residues (commonly K48 or K11), which can form single moieties, poly-ubiquitin branched chains with different lysine linkages (e.g., K63), and/or linear chains on non-lysine residues altogether [42]. The nature of the branched poly-ubiquitin chains and the specific lysine residues on the involved target protein can direct degradation to various cellular outcomes, and ubiquitin itself can be modified into phospho-forms and acetyl-forms, allowing for a broad spectrum of amino acid recycling and cellular control [43]. This versatility suggests ubiquitin-mediated proteolysis as a potent regulator of cellular processes during MRD and thus makes it an important target for miRNA-mediated regulation during hibernation.

In the present study, all ubiquitin- and proteolytic-related terms had negative model coefficients, suggesting a downregulation of these processes during hibernation (Supplementary Files S1–S5). This includes terms that could be linked with alternative cellular outcomes, such as Protein Polyubiquitination (GO BP); likewise, another study on torpid Rickett’s big-footed bat (*Myotis ricketti*) brains saw increased OTUB1, which prevents K48-linked polyubiquitination [44]. Interestingly, another study of skeletal muscle in hibernating *M. lucifugus* measured the upregulation of miR-23a, which has putative targets of TRIM63 and Atrogin1, which prevent muscle-specific protein ubiquitination [17,45,46]. In *S. dauricus*, hindlimb unloading causes mRNA levels of *TRIM63* to increase, whereas, during hibernation, mRNA levels of *TRIM63* remain stable, indicating a protective role for skeletal muscle atrophy [47]. A study on *Murina leucogaster* bats also noted stable levels of TRIM63 and Atrogin1 [48]. This could suggest an MRD-focused suppression of this process as a whole to both conserve cellular energy involved in proteolysis itself and to prevent the breakdown of proteins, which would be energetically expensive to resynthesize during hypometabolism. This is well supported in the literature—hibernators’ lack of skeletal muscle atrophy following hibernation is largely attributed to decreased rates of protein synthesis, balancing out with corresponding decreases in protein degradation [18,49,50,51]. In four tissues of the big brown bat (*Eptesicus fuscus*), the levels of mitochondrial enzyme activities, lactate dehydrogenase isoenzyme content, and lactate dehydrogenase activity did not change between active and hibernation states [52].

This atrophy-linked hypothesis also corresponds to the functional roles of differentially regulated miRNAs observed in Figure 1. For example, miR-221-3p is implicated in skeletal muscle atrophy and maintenance [53] but, intriguingly, is upregulated during hibernation in brown bears (*Ursus arctos*), whereas it is downregulated herein [54]. Contrarily, miR-27b-3p is upregulated in both *U. arctos* and *M. lucifugus* [54]. This miRNA has known regulatory roles in skeletal muscle, specifically mitochondrial function and insulin signaling [55]. Overexpression of miR-27-a/b decreased glucose consumption and glucose uptake; this effect was associated with reduced expression of GLUT4, MAPK 14, and PI3K [56]. miR-27b also targets transcription of myostatin, traditionally regarded as a negative regulator of muscle mass with buildups of myostatin linked with muscle disuse, disease, and/or atrophy [57,58]. In hibernating thirteen-lined ground squirrel (*Ictidomys tridecemlineatus*), the protein levels of muscle myostatin remain steady during hibernation but sharply increase during arousal [58], whereas in *S. dauricus*, levels of myostatin decrease in muscle during early torpor [59]. In another study of *M. lucifugus* skeletal muscle, levels of ten miRNAs were differentially expressed during hibernation. Of these, six miRNAs inhibited myostatin expression either directly or indirectly (downregulation of miR-21 and upregulation of miR-1a-1, miR-206, miR-29b, miR-15-a, and miR-20a) [17]. This lends further credence to the possibility of decreased protein degradation, preventing skeletal muscle atrophy via inhibition of myostatin. Future studies focusing on the potential role of protein degradation in myostatin inhibition are required to elucidate the exact connection between the pathways.

### 4.3. Downregulated Signaling Pathways Tied to Anti-Apoptosis and Atrophy Resistance

A number of signaling pathways were predicted to be downregulated during hibernation as a result of miRNA influence, with GO and KEGG terms corroborating this strong trend. One such signaling pathway is p53 signaling, which had many significant GO terms related to p53 and had negative model coefficients (Figure 1, Figure 2, Figure 3 and Figure 4; Supplementary Files S1–S4), as well as KEGG p53 Signaling Pathway (Figure 5). The p53 protein is a transcription factor that is activated in response to multiple stressors, including hypoxia, heat shock, oxidative stress, and DNA damage, and is regarded as a pro-apoptotic marker due to its ability to induce apoptosis in both a transcription-dependent and independent manner through interactions with Bim, Bak, and the Bcl family [60,61,62,63,64]. A previous study on hibernating *M. lucifugus* skeletal muscle found downregulation of p53 protein expression during hibernation, as well as pro-apoptotic JNK [21]. Therefore, our results reinforce the existing literature that p53 is downregulated for pro-apoptotic suppression, with miRNA likely serving as one such orchestrator of this process.

MAPK signaling may also be differentially regulated via miRNA, with two terms in GO BP and overarching KEGG MAPK Signaling with negative model coefficients (Figure 2 and Figure 5; Supplementary Files S2 and S5). A study on MAPK levels in the hibernating skeletal muscle of *M. lucifugus* found that phospho-active p38 was upregulated, along with the downstream transcription factors ATF-2, CREB, and Elk-1 [22]. Other studies also noted an increase in phospho-HSP27, a downstream factor in the p38 signaling cascade, in both hibernating *M. lucifugus* and *I. tridecemlineatus* [65,66]. This runs counter to our predictions, which suggest an overall downregulation of the MAPK signaling cascade during hibernation. However, this study herein is predicting miRNA influence on pathways and processes of interest during hibernation, which does not take into account the entire cellular environment and the other forms of regulation that occur concurrently with miRNA influence. While miRNAs in their isolation may serve to downregulate MAPK signaling, post-translational modifications, changes in activity, and/or changes in the total protein levels may counter miRNA regulation to activate MAPK signaling instead. Furthermore, the upregulation of MAPK signaling during hibernation in skeletal muscle is not a conserved molecular response. In hibernating Richardson’s ground squirrel (*Spermophilus richardsonii*) skeletal muscle, MAPK activity decreased by 62% during hibernation, despite having no change in the total protein levels of various kinases [67]. Another study on hibernating *I. tridecemlineatus*, which measured the total protein levels of varying MAPK members in the skeletal muscle, displayed an upregulation in p-JNK T183/Y185 [68]. However, a study on *M. lucifugus* muscle measured decreased levels of JNK during hibernation [21]. The measured upregulation of p38, ATF-2, and Elk-1 are at odds with our downstream prediction of reduced p53 signaling. However, phospho-active p38 can also phosphorylate proteins in the MEF2 family, which is shown to be strongly upregulated in the skeletal muscle of hibernating *I. tridecemlineatus* [69]. Therefore, while our predictions indicate that MAPK signaling, in general, is downregulated as a result of miRNA influence, there is much more dynamic variability of individual routes through the network that could be favoring nodes such as p38 and MEF2 while deprioritizing others, including JNK. As such, further studies are needed to elucidate the specificity of MAPK signaling during hibernation in *M. lucifugus* muscle.

A study on hibernating *I. tridecemlineatus* skeletal muscle observed delayed p21 decrease, ERK signaling inhibition, activation of Wnt signaling pathway, and maintenance of low levels of myostatin, all of which are associated with skeletal muscle injury and repair [70]. Some of these results line up with ours, namely, ERK signaling inhibition and myostatin inhibition, as previously discussed. However, the GO BP and KEGG analyses suggest a robust downregulation of canonical and non-canonical Wnt signaling (Figure 2 and Figure 5). This could be a species-specific result, as no research has been done into Wnt signaling in hibernating *M. lucifugus*. However, we cannot dispute other facets of our observed results, which seem to support myostatin inhibition and skeletal muscle atrophy resistance from multiple different molecular routes and which tie into skeletal muscle injury/repair in a manner similar to Andres-Mateos et al.’s study framework [70]. Therefore, since this study is solely on miRNA prediction, it does not take into account the entire cellular environment and other forms of regulation that occur concurrently with miRNA influence. In fact, GO terms relating to other modes of cellular regulation were statistically significant, including Histone Methylation (and two related terms), Peptidyl Lysine Methylation, Histone Acetylation, Histone H3 Acetylation, Protein Acetylation, Protein SUMOlyation, and Histone Ubiquitination (and three related terms) (Figure 2, Figure 3 and Figure 4; Supplementary Files S2–S4). Wnt signaling could, therefore, be dynamically regulated in a manner more complex than our study allows and, by extension, factor Wnt signaling into the skeletal muscle atrophy/repair interplay, as discussed with the other results. Additionally, the skeletal muscle repair likely occurs during interbout arousals, when the animal’s Tb rises to 37 °C for ~1 day to carry out any critical functions before entering another torpor bout [3]. Therefore, further studies quantifying Wnt signaling and other methods of skeletal muscle atrophy/repair during interbout arousal would be prudent to establish a more concrete temporal narrative for these processes.

KEGG pathway analysis showed mTOR signaling as statistically significant with predicted downregulation, along with functionally related insulin signaling pathway (Figure 5). mTOR is at the center of the insulin signaling pathway that regulates protein synthesis and is widely regarded to be at the crux of the protein synthesis/protein degradation balance during hibernation [18]. During hibernation in *I. tridecemlineatus*, mTOR was activated in the liver but suppressed in the skeletal muscle, displaying reduced phospho-mTOR^Ser2448^, along with the upstream regulators p-Akt^Thr473^ and p-TSC2^Thr1462^ and the downstream mTOR targets p-4E-BP1^Thr46^ and p-S6^Ser235^ [71]. In the muscle of *S. dauricus*, Akt-mTOR signaling was suppressed during torpor but increased during interbout arousal [47,72]. To date, a detailed investigation of the mTOR signaling pathway in hibernating *M. lucifugus* has not been conducted, but the already-observed differential regulation of this pathway in other hibernators supports the need for further study to corroborate our findings.

The TGFβ signaling pathway links to a family of growth factors that regulate cellular homeostatic functions, including proliferation/growth, differentiation, migration, and death [73]. Broadly, TGFβ receptors phosphorylate SMAD2 and SMAD3, which translocate into the nucleus after forming a heteromeric complex with SMAD4, and once in the nucleus, the complex regulates gene expression [73,74]. SMAD1, SMAD5, and SMAD8 act in a similar manner but are typically linked with phosphorylation from BMP and INHBE receptors [74]. SMAD6 and SMAD7 are inhibitory SMADs and play a corresponding role [74,75,76]. Four statistically significant terms from GO BP had a negative model coefficient and were directly linked to TGFβ signaling, and the KEGG pathway analysis displayed the TGFβ Signaling Pathway as statistically significant with predicted downregulation as well (Figure 2 and Figure 5; Supplementary Files S2 and S5). There are readily apparent observations to explain this downregulation, from suppressing the energy-expensive nature of this pathway as a whole to suppressing its downstream effects on other such pathways, including cell cycle, MAPK, PI3K, and apoptosis [73]. Another important link from a hibernation perspective involves the connection between TGFβ and myostatin, mentioned above as a negative regulator of muscle mass. Treatment with TGFβ and/or overexpression of SMAD-2, -3, or -4 results in a significant increase in the myostatin promoter activity, and the myostatin promoter region contains a binding site for the phospho-SMAD heteromeric complex; thus, SMAD2 is unequivocally a critical regulator both downstream of the receptor–myostatin complex and upstream of myostatin expression [58,77]. Decreases in TGFβ signaling would lead to corresponding decreases in myostatin expression, in turn preventing skeletal muscle atrophy during hibernation. Indeed, studies have shown that myostatin inhibition decreases fibrosis after muscle injury, and downregulation of myostatin, TGFβ1, and pro-inflammatory cytokines play a role in the lack of tissue fibrosis seen in the injured hibernating squirrel muscles [70,78]. The phosphorylation of SMAD2/3 is reduced in the torpid muscle in *S. dauricus* [59], while the BMP signaling maintenance/TGFβ inhibition has been proposed as a mechanism of atrophy resistance in the hibernating *U. arctos* [79]. Taken together, all of these results further corroborate the hypothesized functional roles of decreased protein degradation and atrophy resistance, as discussed earlier; however, additional confirmatory studies are needed.

### 4.4. Downregulated Cytoskeletal Processes May Link to ARP3-Mediated Insulin Signaling

A number of GO terms predicted to be differentially regulated during hibernation are also related to cytoskeletal processes and subcomponents: Cytoskeleton Organization, Cell-Cell Adhesion, and Biological Adhesion (GO BP); Cell Substrate Junction and Anchoring Junction (GO CC); and Focal Adhesion, Cell Adhesion Molecules CAMS, Regulation of Actin Cytoskeleton, Adherens Junction, Focal Adhesion, and Gap Junction (KEGG) (Figure 2, Figure 3, Figure 4 and Figure 5; Supplementary Files S2–S5). Unlike the other trends observed thus far, many of these terms were differentially regulated, with predicted upregulation of Cell Adhesion Molecules, Intermediate Filament, Cell-Cell Signaling, and Cell-Cell Adhesion and downregulation of Cytoskeletal Organization, Cytoskeleton, Cytoskeletal Part, Microtubule Cytoskeleton, Cell Substrate Junction, Anchoring Junction, Microtubule, Cytoskeletal Protein Binding, Focal Adhesion, and Adherens Junction to name a few (Figure 5; Supplementary File S5). However, some of these GO terms may be overlapping with other downregulated GO terms and causing artificial downregulation by proxy, e.g., microtubule-related cytoskeleton with cell cycle and kinetochore-linked processes (Figure 2; Supplementary File S2).

The studies on hibernators demonstrate that skeletal muscle cytoskeletal ultrastructure is well-maintained and does not undergo substantial degradation during hibernation [80]. While little research has been conducted on cytoskeletal features of *M. lucifugus* specifically, a proteomics study was conducted on the torpid brains of hibernating *M. ricketti*, which displayed differential regulation of 24 proteins involved in reduced cytoskeletal plasticity and increased vesicle-related proteins [44]. Specifically, suppression in the growth and remodeling of branched-chain actin filaments during torpor was suggested via interactions among Arp2/3, CFL1, and CAPZB [44]. Indeed, GO Regulation of ARP2/3 Complex Mediated Actin Nucleation was a statistically significant term in GO BP with a negative model coefficient herein (Supplementary File S2). KLC2, which transports cargo towards the plus end of microtubules, was also shown to have significantly decreased expression [44]. An isoform of TPT1 that maintains microtubule stabilization and an isoform of CCT1 that aids in actin and tubulin folding also showed decreased protein expression [44]. Furthermore, the expression of the intermediate filament proteins PALM and NEFL also decreased [44]. However, nine GO BP terms herein and 12 in GO CC related to vesicle transport were statistically significant but had negative model coefficients (Figure 2 and Figure 3; Supplementary Files S2 and S3). Since some of them were Golgi-transport-based, it is possible that this downregulation falls under the umbrella of protein synthesis, which is largely reduced during MRD. Moreover, this discrepancy could be due to tissue-specific functional roles during hibernation.

Another compelling hypothesis centers around the fact that ARP3 is also responsible for insulin-stimulated GLUT4 translocation to the surface of muscle cells [81]. More specifically, insulin activates PI3K-dependent Rac GTPase, which leads to actin remodeling for GLUT4 translocation to the surface of muscle cells [82,83,84]. ARP3 exists as a downstream effector of Rac GTPase, and siRNA-mediated silencing of ARP3 cripples the insulin-induced actin remodeling and, by extension, GLUT4 translocation [81,85]. Our results support this—in GO BP, the Positive Regulation of Glucose Import in Response to Insulin Stimulus, Cellular Response to Insulin Stimulus, Response to Insulin, Insulin Receptor Signaling Pathway had negative model coefficients as well as KEGG Insulin Signaling Pathway, insinuating downregulation of this collective process via miRNA (Figure 2 and Figure 5; Supplementary Files S2 and S5). This provides an intriguing possibility for miRNA-dependent suppression of insulin signaling via ARP3 actin remodeling and also links to our observed downregulation in vesicle trafficking GO terms.

Overall, while it appears that miRNA may be downregulating some cell motility and vesicle trafficking processes, these have considerable overlap with other more prominent downregulated pathways, including cell cycle and protein synthesis. A more specific function may link to downregulated insulin signaling via ARP3-mediated dysregulation of actin remodeling during hibernation. Further research will need to be performed into cytoskeletal remodeling during hibernation in *M. lucifugus* to validate and expand upon these findings, including a potential link with ARP2/3 suppression.

## 5. Conclusions

This study found 11 differentially regulated miRNAs whose expression changed in torpid *M. lucifugus*. The gene set analysis predicted that cell cycle processes were highly suppressed, which is well-represented in the literature as a global MRD hallmark. The ubiquitin-mediated proteolysis was also predicted to be downregulated, which could counteract skeletal muscle atrophy via myostatin inhibition, especially since differentially regulated miR-27b directly targets myostatin. The suppression of protein degradation may also balance the corresponding decrease in protein synthesis via mTOR, which was indeed observed alongside a collective suppression of signaling pathways, including MAPK, p53, mTOR, and TGFβ. Intriguingly, TGFβ also ties into our model of skeletal muscle atrophy resistance since SMAD2 is a key negative regulator of myostatin. Finally, the downregulation of cytoskeletal and vesicle trafficking may suppress insulin signaling via the ARP3-mediated actin remodeling while also supplementing the cell cycle and mTOR/protein synthesis downregulation. Taken together, our results indicate that miRNA regulation corroborates both conserved MRD survival strategies and more hibernation- and tissue-specific roles, including skeletal muscle atrophy resistance and insulin signaling suppression. Further research will expand our knowledge of hibernating skeletal muscle atrophy resistance, as well as *M. lucifugus* as a specific biological model.

## Data Availability

Raw sequencing data can be found on the Sequence Read Archive database (BioProject ID: PRJNA1100517).

## Supplementary Materials

The following supporting information can be downloaded at: https://www.mdpi.com/article/10.3390/cells13242074/s1, File S1: Differential Expression; File S2: GO Biological Processes; File S3: GO Cellular Components; File S4: GO Molecular Functions; File S5: KEGG; File S6: MicroRNA Target Genes.
